# Selected Nutrition and Management Strategies in Suckling Pigs to Improve Post-Weaning Outcomes

**DOI:** 10.3390/ani13121998

**Published:** 2023-06-15

**Authors:** Elisa A. Arnaud, Gillian E. Gardiner, Peadar G. Lawlor

**Affiliations:** 1 Teagasc Pig Development Department, Animal and Grassland Research and Innovation Centre, Moorepark, Fermoy, P61 C996 County Cork, Ireland; elisa.arnaud@teagasc.ie; 2 Eco-Innovation Research Centre, Department of Science, Waterford Campus, South East Technological University, X91 K0EK Waterford, Ireland; gillian.gardiner@setu.ie

**Keywords:** weaning, large litters, pain relief, split-suckling, enzymes, L-glutamine, creep feed

## Abstract

**Simple Summary:**

Weaning involves the removal of piglets from a sow, and on commercial farms, it usually occurs at between 3 and 5 weeks of age. Newly weaned piglets face physical, social, environmental, management and dietary challenges. Consequently, post-weaning, they often experience reduced feed intake, poor growth and, in some cases, diarrhoea. There are many interventions which can be employed on-farm during the suckling period to ease the transition at weaning for piglets. Strategies such as supervised farrowing, post-farrowing pain relief for sows, the use of nurse sows, cross-fostering, the administration of energy supplements/feed additives to piglets and liquid/dry creep feeding have been investigated. The objective of these strategies is to promote earlier feed exploration, increase early post-weaning feed intake and growth and improve intestinal maturity. This review focuses in particular on pain relief for sows, piglet management at birth, and the provision of supplementary milk/liquid feed and feed additives to suckling piglets. Carefully selected, these strategies have the potential to increase the lifetime growth and health of pigs from large litters.

**Abstract:**

Weaning is a critical period in a pig’s life. Piglets are confronted with abrupt changes to their physical and social environment, as well as management and nutritional changes. Weaning has always been associated with a growth check and is frequently accompanied by post-weaning diarrhoea in piglets. However, rapid increases in litter size in the last decade have increased within-litter piglet weight variation, with piglets now generally lighter at weaning, making the challenges associated with weaning even greater. Many interventions can be employed during the suckling period to ease the weaning transition for piglets. Pre-weaning strategies such as supervised farrowing (assistance with suckling and oxytocin provision), the provision of pain relief to sows around farrowing, split-suckling, early oral supplementation with glucose, bovine colostrum, faecal microbiota transplantation, feed additives and solid and liquid creep feeding (milk and liquid feed) have all been investigated. The objective of these strategies is to stimulate earlier maturation of the digestive tract, improve immunity, reduce latency to the first feed post-weaning and increase early post-weaning feed intake and growth. This review focuses in particular on: (1) pain relief provision to sows around farrowing, (2)split-suckling of piglets, (3) pre-weaning provision of supplementary milk and/or liquid feed, (4) other strategies to stimulate earlier enzyme production (e.g., enzyme supplementation), (5) other nutritional strategies to promote improved gut structure and function (e.g., L-glutamine supplementation), and (6) other strategies to modulate gut microbiota (e.g., probiotics and prebiotics). Correctly implementing these strategies can, not only increase post-weaning growth and reduce mortality, but also maximise lifetime growth in pigs.

## 1. Introduction

Weaning is a critical period in pigs’ lives, during which, they have to cope with many changes in their physical and social environment, as well as in their management and nutrition. They are separated from their mother, and piglets from different litters are mixed together, often resulting in fighting. As a result, increased levels of cortisol are often observed in pigs at weaning, indicating increased stress [1]. Pigs also come into contact with ‘new’ microorganisms which can impact their health. The diet is also modified; up to weaning, pigs suckle ~20 small meals of milk each day, which is high in fat and lactose, and at weaning, this is normally replaced by large meals of a less digestible, plant-based, solid diet [2,3]. The physiological changes associated with weaning have been described in several reviews [4,5] and so will not be discussed here. All of these changes/stresses often lead to a reduction in post-weaning feed intake and weight gain (commonly referred to as a post-weaning ‘growth check’) [3] and intestinal dysbiosis [6]. The extent of this ‘growth check’ and dysbiosis depends on how rapidly the pig is able to adapt to its new circumstances. Intestinal dysbiosis is one of the leading factors contributing to post-weaning diarrhoea (PWD) [6]. As reviewed by Canibe et al. [7], PWD is a widespread disease that has major consequences for productivity and mortality on pig farms. Until recently, pharmacological doses of zinc oxide (ZnO; 2500 ppm of zinc) were widely included in the diet during the 2 first weeks post-weaning to prevent PWD and in-feed antibiotics were also used. However, antimicrobial resistance (AMR) has been linked with antibiotic and ZnO use in pigs [8]. The emergence of antibiotic-resistant bacteria in pigs is considered a major risk for public health, as resistant organisms can spread from pigs to humans, limiting the number of effective antibiotics available to treat human disease [9]. Therefore, in 2022, in response to this rise in AMR, the European Union prohibited all forms of routine antibiotic use in farming, including preventive group treatments and the use of medicated feed for prophylaxis [10], and banned the use of pharmacological levels of ZnO in pigs [11].

Intensive genetic selection has led to hyperprolific sows that give birth to more piglets than the number of functional teats available on the sow [12]. This increase in litter size has led to more heterogenous litters, with a lower average weight at birth and a higher proportion of ‘light’ piglets born alive. Furthermore, although the demand for colostrum and milk by the litter increases with increasing litter size, sows have a finite ability to produce both [13]. Therefore, the mean volumes of colostrum and milk available for individual pigs decrease as litter size increases. This is important, as piglets need to receive at least 200 g of colostrum within the first 24 h of life to survive [14], and milk consumption is directly correlated with pre-weaning growth. Furthermore, achieving a high weaning weight is key to limiting the growth check at weaning [3] and increasing lifetime growth [15].

Several recent reviews discuss the different strategies that can be used pre-weaning to address the challenges outlined above (see Table 1). Rather than duplicating the content of these reviews, this review will focus on the following areas: (1) post-farrowing pain relief provision to sows, (2) split-suckling of piglets, (3) pre-weaning provision of supplementary milk and/or liquid feed, (4) strategies to stimulate earlier enzyme production in the intestine (e.g., extraneous enzyme supplementation), (5) strategies to stimulate gut structure and function (e.g., supplementing piglets with L-glutamine) and (6) strategies to modulate gut microbiota (e.g., pro-, pre- and post-biotics). These areas have not been reviewed recently. Correctly implementing these strategies can, not only increase post-weaning growth and reduce mortality, but also maximise lifetime growth in pigs.

## 2. Management Strategies in Sows and Suckling Pigs to Increase Colostrum Intake

A large litter size has consequences for uterine capacity and the post-natal life experience of piglets [27]. This review will focus on the latter. Piglets need to receive at least 200 g of colostrum within the first 24 h of life to survive [14], and 250 g to ensure optimal growth [28]. In large litters, some piglets often fail to ingest a sufficient quantity of colostrum during the first 24 h. This is critical as colostrum contains immunoglobulins, 80% of which are immunoglobulin G (IgG), which are of primary importance for the transfer of passive immunity from the sow to the piglets [29]. Colostrum also provides energy to new-born piglets, as pigs have low energy reserves when born [30]. It contains other biological components of importance for pig development and health, such as leukocytes [30] and various growth factors [31]. It also has laxative properties which are essential in helping to eliminate the first stool. Colostrum quality decreases rapidly during the first 24 h post-partum [32], with the highest immunoglobulin concentrations found within the first 4 h post-partum [2]. If the optimal quantity of colostrum ingested per piglet during the first 24 h of life is set at 250 g [32], and given an average number of piglets born alive per litter of 15, a nursing sow needs to produce at least 3.75 kg of colostrum within the first day post-partum. From data collected from an experimental herd, Quesnel et al. [32] estimated that among 200 sows, 35% do not even produce the 3.25 kg of colostrum required to fulfil the needs of 13 piglets. Therefore, considering the importance of achieving an adequate intake of colostrum for the development and health of the pig, it is necessary to implement strategies to ensure that each pig within a litter receives an equal and adequate share of the colostrum available within the first 24 h of birth. Such strategies are discussed below.

### 2.1. Pain Management in Sows

Piglets commence suckling their sow within minutes after their birth. The sow must be comfortable in order to facilitate suckling by her litter so that piglets consume adequate quantities of colostrum and then milk. If a sow lies quietly, it is assumed that piglets have ready access to the udder and therefore unlimited access to colostrum and milk [33]. It is generally accepted that parturition is a painful process and that post-farrowing pain and inflammation can impede the sow’s ability to nurse. Farrowing leads to both visceral pain [17] (e.g., ‘pain from the inner organs including pain manifested at the udder and dependent on the conduction of pain information through activation of visceral afferent fibres’ [34]) and somatic pain [17] (e.g., ‘pain arising from damaged skin, joints bones or muscles and dependent on activation of somatic afferent fibres’ [34]). In sows, inflammatory damage can still be observed one week after farrowing, as demonstrated by high levels of C-reactive protein and haptoglobin in the blood [35]. Several factors can impact the degree of inflammation and pain caused by the farrowing process, such as prolonged farrowing duration and parturition difficulties, also referred to as dystocia [17].

Providing non-steroidal anti-inflammatory drugs (NSAIDs) and NSAID-like drugs to the sow around farrowing can alleviate the associated pain in the sow and therefore increase her receptiveness to suckling by her piglets. As reviewed by Schoos et al. [16], NSAIDs have antipyretic, analgesic and anti-inflammatory effects, while NSAID-like drugs have only antipyretic and analgesic effects. To our knowledge, in 2023, there were five NSAIDs (meloxicam, flunixin, tolfenamic acid, ketoprofen and sodium salicylic acid) and two NSAID-like drugs (paracetamol and metamizole) authorised by the European Medicines Agency for use in pigs [36]. Most NSAIDs act by inhibiting the enzymes cyclo-oxygenase 1 (COX-1) and 2 (COX-2). Some of them, such as meloxicam, are selective COX-2 inhibitors. Cyclo-oxygenases are involved in the conversion of arachidonic acid into thromboxanes, prostaglandins and prostacyclins, which have a role in platelet adhesion, vasodilation, antinociception and body temperature set-point determined in the hypothalamus [37]. Cyclo-oxygenase 1 is always expressed in the body and plays a role in maintaining gastrointestinal mucosal integrity, whereas COX-2 is only expressed during an inflammatory response [37]. Therefore, selective COX-2 inhibitors, such as meloxicam, when administered to provide post-partum pain relief, provide the required anti-inflammatory benefits without compromising intestinal mucosa integrity [37,38].

Table 2 summarises the findings of studies that have used NSAIDs or NSAID-like drugs in sows around the periparturient period. NSAIDs or NSAID-like drugs can be administered orally via gavage [39,40,41], intramuscularly [42,43,44,45,46,47,48] or orally with feed [49]. In general, the timing of administration ranges from 1.5 h [42,48] to 12 h post-partum [43,45,47] for intramuscular injections in healthy sows. When given orally, the drug was provided at the beginning of the farrowing process in two studies [39,40]. In two other studies, oral administration started 2 to 3 days before parturition and was repeated daily for up to 4 days post-partum [41,49]. The results from these studies indicate that the use of NSAID drugs can benefit both the sow [44,46,49] and the piglets [39,40,47]. Meloxicam does not reduce fever in sows post-partum [42,43], while flunixin [44] and ketoprofen [45] do. Ketoprofen [46] and paracetamol [49] reduced the back fat loss experienced by sows during lactation. The reduction in back fat loss can be explained by the decrease in feed refusal in sows treated with these drugs during the treatment period [46]. When administered orally at the beginning of farrowing, meloxicam positively influences piglet growth during lactation, but it does not seem to affect piglet mortality [39,40]. On the contrary, when administered intramuscularly after parturition, ketoprofen reduced mortality [45,47] but failed to demonstrate a positive growth effect [45,46]. Few studies compared NSAIDs or NSAID-like drugs within the same study [41,44,50]. However, Schoos et al. [41] treated sows suffering from postpartum dysgalactia syndrome (PDS) with either meloxicam or paracetamol. Rectal temperature, piglet mortality and growth were not affected by either treatment compared to control untreated sows. However, the rectal temperature was lower in sows treated with paracetamol compared with meloxicam [41]. Likewise, Hirsch et al. [50] did not observe any differences between meloxicam and flunixin regarding their ability to reduce clinical signs of mastitis–metritis–agalactia syndrome. However, in this study, mortality was lower in piglets born to meloxicam-treated sows compared with those treated with flunixin [50]. Comparing flunixin to metamizole administration, rectal temperature was lower in sows treated with flunixin [44]. Post-partum NSAID administration to sows can also increase immunoglobulin transfer from sows to piglets. Higher levels of IgG [39] and IgA [40] were observed on day 1 after birth in the serum of piglets suckling sows supplemented with meloxicam. In the study by Navarro et al. [40], the higher level of IgA persisted until day 9 after birth. The better immunity acquired by piglets from sows which received meloxicam could explain the better growth experienced by those pigs.

To our knowledge, apart from one study from our group, there are no published studies investigating the effects of NSAID provision to sows on pig growth post-weaning. Our study ) showed that providing meloxicam to sows within 2 h post-partum can increase pig weaning weight by 4.5% and slaughter weight by 3.1% [51]. Meloxicam administration to sows also reduced the volume of antibiotics and anti-inflammatories administered to piglets pre-weaning and tended to increase colostrum intake in piglets. This increased transfer of passive immunity to suckling piglets could explain the reduction in pre-weaning medication usage observed, as well as the increased growth to weaning. Feed efficiency was improved during the first week after weaning in pigs from the meloxicam group. Heavier weaning weight and the better feed efficiency at weaning likely explain the observed increase in slaughter weight [51].

While it is evident that many of the NSAID and NSAID-like drugs, when administered around farrowing, can confer benefits to both sows and piglets, there is no clear consensus on the drug of choice to use. However, as meloxicam can benefit serum immunoglobulin status in piglets and increase piglet survivability and growth in suckling and weaned pigs, it might be particularly beneficial. From these studies, it appears that NSAIDs and NSAID-like drugs should be provided within 2 h post-partum to the sow when administered intramuscularly and at the beginning of farrowing when given via oral gavage.

### 2.2. Split-Suckling

Cross fostering is sometimes used on-farm to help increase colostrum intake in piglets. However, it seems that only maternally-derived cells can cross the gut barrier in the neonate and that cells from a foster mother’s colostrum are not well absorbed by cross-fostered piglets [52]. Additionally, cross-fostering is usually conducted too late after farrowing (normally after 24 h), and while it will help to ensure milk intake for all pigs, it does little to increase colostrum intake. Contrary to this, split-suckling can help to ensure that all piglets within large litters get a chance to suckle and therefore consume sufficient colostrum during the critical early post-partum window. Split-suckling is defined as the removal of part of the litter from the sow for a set period of time to allow the remaining piglets to suckle the sow without competition [53]. The strategy is particularly useful when the number of piglets born alive per sow is high, exceeding the number of functional teats available on the sow, and where fostering options are limited [54]. Split-suckling should allow all piglets to access colostrum, and thereby to acquire passive immunity [54] and sufficient energy immediately after birth. However, there is no consensus on how to apply split-suckling in terms of its duration and timing, the number of piglets removed and the number of piglets left on the sow, and the category (weight, birth order, etc.) of piglets to remove [55]. The litter can be split by removing only the heaviest piglets for a period of time while leaving the lightest piglets to suckle [56,57]. Another way to apply split-suckling is to take into consideration birth order and to remove the first-born piglets, giving the piglets born later time to access colostrum [58]. It might also be interesting to assess the litter and remove piglets with a ‘full’ belly (which have already ingested colostrum) regardless of weight or birth order. The length of time piglets are removed from the udder varies across studies [55,58,59]. However, it is generally recommended to leave the first group of pigs for 1 h at the udder before placing back the piglets which were removed The first day of life is the most critical period to apply split-suckling, as the quantity and quality of colostrum decreases rapidly during the first 24 h following parturition [32]. However, as most piglet mortality occurs during the first 3 days of life [60], some propose the application of split-suckling until day 3 post-partum, alternating groups every 3 h for 12 h per day [55]. In addition, a study from Donovan and Dritz [57], in which ADG within litters was more homogeneous in litters with more than nine pigs born alive, suggests that split-suckling is only beneficial in large litters.

As outlined above, there is no consensus on the best split-suckling strategy to apply, and results vary considerably between studies. Muns et al. [59] failed to demonstrate beneficial outcomes in terms of piglet growth and survival when they removed the heaviest piglets (>1.30 kg) for 2 h within the first 24 h after birth [59]. However, other authors reported increased piglet growth [58] and increased survival of small piglets [61] when split-suckling was applied during the 1st day after birth. In one of these studies, Morton et al. [58] reported a ~17% increase in piglet growth up to day 7 post-partum for all pigs when the six heaviest piglets were removed from the udder for 1.5 h, leaving the remainder of the litter to suckle without competition during this time window. Huser et al. [61] reported a 13% increase in the pre-weaning survival rate for small piglets in litters where heavy piglets (>2.08 kg) were removed once for 2 h in the morning following farrowing.

The timing and duration of the split-suckling bout and how many times split-suckling bouts are conducted will all influence the success observed with split-suckling. For example, Vandaele et al. [55] conducted split-suckling during the first 3 days of life by alternating two groups of the heavier piglets at the udder every 3 h for 12 h per day, while the smallest piglets always remained with the sow, the aim being to provide them with a nursing advantage [55]. However, this split-suckling strategy reduced the growth of all pigs and did not increase the colostrum intake or survival of the smallest piglets [55]. To our knowledge, there is no published study investigating the effects of split-suckling on pig growth post-weaning. One study from our group [51] showed that split-suckling by removing the six heaviest piglets for two periods of 1.5 h starting 4 h after farrowing onset reduces average pig weaning weight by 2.1% and slaughter weight by 1.5%.

Based on the above, it would seem important for the success of split-suckling that it is conducted within the first 24 h of the piglet’s life, that piglets are not removed from their sow for more than 2 h during split-suckling bouts and that they are removed only once. Perhaps an attempt should be made to determine if individual pigs have suckled colostrum and are full, and then only those pigs that are full should be removed. In the case of successful split-suckling strategies, increases in piglet growth up to weaning could lead to improved post-weaning growth, as weaning weight is highly correlated to subsequent lifetime growth in pigs [15].

## 3. Nutritional Strategies in Suckling Pigs to Improve Growth and Intestinal Maturity at Weaning

### 3.1. Strategies to Help Maximise Dry Matter Intake in Piglets Prior to Weaning

Pre-weaning strategies that effectively increase nutrient intake and growth in piglets up to weaning are important since weaning weight is positively correlated with subsequent health and growth in pigs [15]. Increasing weaning weight and ensuring good intestinal health at weaning can help pigs to overcome the normal stresses associated with weaning. Creep feeding suckling piglets with dry feed, liquid milk replacer and/or liquid feed are strategies which can help increase pre-weaning dry matter intake (DMI) and consequently growth, resulting in heavier pigs at weaning.

#### 3.1.1. Provision of Solid Creep Feed Pre-Weaning

The provision of dry creep feed to suckling piglets is a common practice which has previously been well reviewed [22,26]. Creep feed provision has the primary objective of supporting sow milk production, as this becomes a limiting factor for piglet growth during mid-lactation, especially in large litters [26]. In addition, providing dry creep feed to suckling piglets can help to habituate them to solid feed prior to weaning, increase feed intake and growth and improve intestinal structure and function post-weaning. The effects of dry creep feeding on pre- and post-weaning growth are not always consistent; some studies find the practice beneficial, while others do not (reviewed by Tokach et al. [26]). Inconsistencies in the response to the dry creep feeding of suckling piglets can be explained by the different approaches to creep feed provision taken by the authors. The duration of creep feeding and piglet age at weaning were reviewed by Tokach et al. [26] as two important factors affecting the response to creep feeding. Studies in which litters were weaned at 35 days of age or greater demonstrated a consistent gain in weaning weight with creep feeding [26], which was most likely due to increased creep feed consumption with increasing weaning age. Creep feeding can start as early as two days of age to as late as a couple of days before weaning [26]. The percentage of piglets within each litter eating creep feed (i.e., piglets considered as “eaters”) can also explain differences in outcomes between studies, with litters with a higher proportion of ‘eaters’ benefitting most from creep feeding. Several factors can affect the creep feed intake of individual pigs, such as the availability of the sow’s milk (e.g., if the pig has access to a teat producing a low quantity of milk), the piglet’s birth weight, the size of the pellets provided, the creep feeding duration, the composition of the creep feed itself and its accessibility and organoleptic properties (as reviewed by Huting et al. [22] and Tokach et al. [26]). The use of flavours in dry creep feed has also been well reviewed to date, and it would appear that there is a lack of effect on creep feed intake and pig growth, most likely due to variable palatability preferences and perceptions between piglets [26]. Providing dry creep feed can help to develop the intestinal tract so that it can cope better with the post-weaning diet, and this is a principal benefit of the practice. It may stimulate earlier enzyme secretory capacity in the gastrointestinal tract (GIT) of piglets, thereby enabling the digestion of non-milk ingredients normally found in diets after weaning. The effects of providing dry creep feed on gut structure are not always consistent either (as reviewed by Huting et al. [22]). In order to obtain the greatest benefit, it is generally accepted that creep feed should be offered in small amounts to avoid feed wastage and to keep the feed as fresh as possible. Creep feed supplementation should be started on day 7 to 10 of age to maximise intake. In practice, creep feed intakes can be very variable. Therefore, providing creep feed in liquid form might be a solution to promote intake.

#### 3.1.2. Provision of Supplemental Milk Pre-Weaning

**Figure 1:**
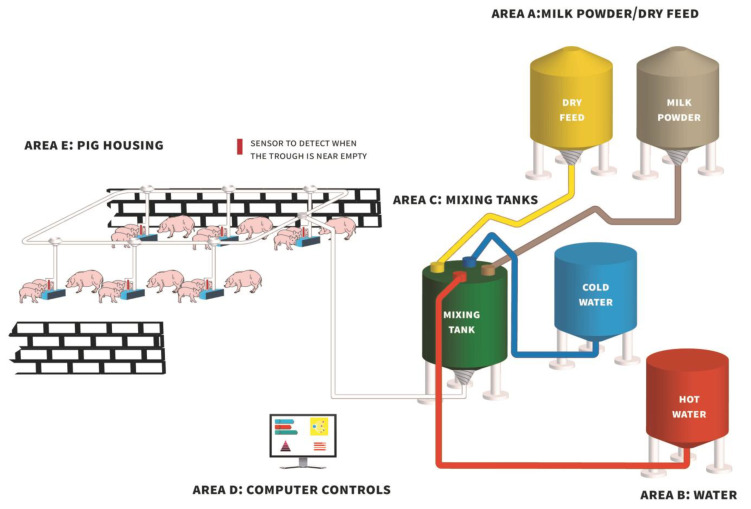
Diagram of a typical automated liquid milk/feed delivery system (based on the Babyfeed system; Schauer Agrotronic GmbH, Prambachkirchen, Austria) demonstrating how milk powder/dry feed from feed bins (Area A) and water (Area B) are delivered to a central mixing tank (Area C) and agitated, followed by the delivery of liquid milk/feed to farrowing pens via a series of pipes for consumption by piglets (Area E).

Providing piglets with a liquid diet (supplementary milk or a diet mixed with milk/water) pre-weaning could be a promising strategy to increase creep feed intake prior to weaning. This strategy could reduce the feed neophobia experienced by suckling piglets toward solid feed, increasing DMI and the number of eaters, thereby positively influencing weaning weight [62] and post-weaning growth [63]. The provision of a supplementary milk replacer to suckling piglets enables the rearing of large litters while they continue suckling their mother [64]. However, there is no consensus regarding when milk replacer provision should commence, how often milk replacer should be offered during the day and for how long during lactation the practice should be implemented. Supplemental milk can be provided during the entire lactation period starting from 24 h after farrowing [65,66] or for a shorter amount of time starting 5 to 10 days before weaning [67,68]. Milk can be supplemented ad libitum [63,64], or access to milk may be restricted to a set period of time each day [62,69]. Milk can be prepared and fed to piglets manually or through an automated delivery system. Automated delivery systems for supplemental milk replacer are now quite common on European farms (see schematic, Figure 1). These systems mix milk replacer powder with warm water at a pre-determined concentration. The feeding frequency can be set to approximate ad libitum feeding. Usually, fresh milk is prepared at least twice daily. Where milk cups are used, the cups contain a push lever which piglets use to release milk [70] and milk is available on demand (e.g., Neopigg^TM^ RescueCare system by Cargill, United States; CulinaCup by Big Dutchman, Germany). Alternatively, some systems contain sensors within troughs, and when the milk in the trough is below the level of the sensor, fresh milk is delivered to that trough at the next pre-determined feeding time (e.g., Babyfeed system by Schauer Agrotronic GmbH, Austria; CulinaFlex by Big Dutchman, Germany). Regardless of the milk feeding system type, it is essential for the system to be hygienic; systems are normally flushed with an acid daily and an alkaline detergent flush is performed once per week, but sometimes as infrequently as once a month. This is carried out to minimise biofilm formation and to help to ensure good microbial quality in the milk. By cleaning with peracetic acid daily and using an alkaline detergent once a month, Pustal et al. [64] did not observe any increases in bacterial counts in milk sampled from the tank at the end of the day every 5 days from the 3rd to the 23rd day of supplementation. However, bacterial growth was not monitored in the tank during the day.

Table 3 summarises the effect of milk replacer supplementation to suckling litters on pre-weaning and post-weaning piglet growth and health. Milk intakes are very variable within and between litters. Intakes can be influenced by several factors, such as room temperature or the quantity of milk produced by the sow (as reviewed by Huting et al. [22]). Several studies showed an increase in pre-weaning ADG and weaning weight when suckling piglets were supplemented with liquid milk [62,63,67,68,71]. Wolter et al. [63] observed a 16% increase in weaning weight when piglets were supplemented with liquid milk from day 3 post-farrowing to day 21 (weaning). This observation was confirmed in a study conducted by De Greeff et al. [62] in which piglets supplemented with milk from day 2 to 21 (weaning) were 8% heavier at weaning. Contrary to this, Pustal et al. [64] failed to find an increase in pre-weaning piglet ADG and weaning weight when supplementary milk was provided to piglets from day 2 post-farrowing to day 28 (weaning). However, in the latter study, milk-supplemented litters weaned 1.1 piglets more than unsupplemented litters, and the litter weaning weight was increased as a consequence. Wolter et al. [63] also found that pre-weaning milk supplementation increased the number of pigs weaned by 0.5 piglets per litter. The effect of milk supplementation on pre-weaning mortality is variable, with some studies finding a reduction in pre-weaning mortality when piglets were supplemented with milk [63,69] and others showing no effect [65,66,72]. Relatively few studies have followed the growth of milk-supplemented piglets into the post-weaning period and beyond. Wolter et al. [63] did not observe any effects of pre-weaning milk supplementation on pig growth immediately post-weaning (from weaning to 25 kg). However, they found that pigs supplemented with milk replacer pre-weaning had a 4.5% increase in average daily feed intake (ADFI) and tended to have an increase in ADG (of 3.3%) during the middle of the grower period (from 25 to 65 kg body weight (BW)). As a consequence, supplemented pigs reached the target slaughter weight (110 kg) 3 days before their non-supplemented counterparts [63]. Park et al. [69] also monitored the post-weaning growth of pigs that had been provided with supplementary milk from day 4 after birth up to weaning on day 21. In an experiment conducted in the autumn, weaning weight and pre-weaning mortality were not influenced by milk provision to suckling pigs. In another conducted in July, weaning weight was increased and pre-weaning mortality reduced in pigs supplemented with milk replacer. Therefore, the prevailing temperatures during the period in which pre-weaning milk supplementation to suckling litters is performed may influence intake of supplemental milk and therefore the response observed. This is possibly because of reduced milk production in sows due to the reduced lactation feed intake normally observed during periods of high temperature. However, in the latter study, there was no effect on the final slaughter weight in either experiment. Overall, providing supplementary milk can be an effective strategy to increase creep feed intake prior to weaning. However, there is a possibility that it could reduce the consumption of sow milk by piglets. Nevertheless, based on a study from our group, sow weight and back fat changes during lactation were not affected when supplemental milk was provided to suckling piglets (Arnaud et al. [73], unpublished). Based on these results, it would appear that this strategy did not benefit the sow, and hence, that providing supplemental milk did not reduce suckling in piglets. However, the litter size was high (~16 piglets born alive), and we believe that this is when the provision of supplementary milk to suckling pigs is particularly beneficial.

Few studies have investigated the effect of supplementing milk replacer pre-weaning on gut maturity at weaning. De Greeff et al. [62] observed a 26% increase in small intestinal weight in suckling piglets supplemented with milk replacer for 21 days, as well as a higher relative weight:length ratio compared with control non-supplemented piglets, indicating that the milk supplement stimulated intestinal growth. These authors also observed an increase in crypt depth and a lower villus height:crypt depth ratio in the ileum of milk-supplemented piglets on day 21 (weaning). This indication of higher cell-proliferation rates could imply an impairment of intestinal integrity in this study. However, Hu et al. [74] did not observe any differences in villus height or crypt depth in the jejunum on day 28 (weaning) and day 35 (8 days post-weaning) in pigs supplemented with milk during the suckling period compared to non-supplemented pigs. Regarding enzyme production, they found lower lactase and higher sucrase activity on day 28 and higher maltase activity on day 35 in the jejunum of pigs supplemented with milk, suggesting that pre-weaning supplementary milk provision to suckling pigs may induce earlier maturation of the jejunum. The effect of pre-weaning liquid milk supplementation on intestinal microbiota composition is not consistent, with some studies demonstrating a benefit from pre-weaning milk supplementation [74] and others not [75]. Hu et al. [74] observed a greater abundance of *Clostridium XI*, *Turicibacter* and *Moraxella* at 28 days of age in the jejunum of piglets supplemented with milk from day 4 to 28 after birth in comparison to control unsupplemented pigs. In addition, they demonstrated an increased abundance of *Lactobacillus* and a decreased abundance of *Streptococcus* and *Blautia* in the jejunum on day 35 (7 days post-weaning), indicating that the milk supplement may have increased the abundance of beneficial bacteria in the small intestine, therefore helping to maintain intestinal homeostasis. In a study in which pigs were supplemented with milk from day 7 post-partum until day 21 (weaning), Jin et al. [75] observed that the supplemented group had higher bacterial species richness estimates (ACE and Chao1) in the jejunum compared to control unsupplemented pigs, indicating a higher number of bacterial species. However, the supplemented group had similar Simpson and Shannon diversity indices compared to the control, indicating that there were no differences in the abundance of each species. The supplemented group had lower abundances of *Romboutsia*, *Actinobacillus*, *Bacteroides* and *Lactobacillus* than the control group, indicating that the abundance of some beneficial bacteria (such as *Lactobacillus)* was reduced in pigs supplemented with milk. The authors surmise that the decrease in *Lactobacillus* abundance could be the result of reduced ingestion of sow milk containing oligosaccharides. However, this would also have been the case in the study by Hu et al. [74], and they observed the opposite. This lack of agreement across studies is likely due to differences in the composition of the supplementary milk fed. De Greeff et al. [62] observed an increase in concentrations of the volatile fatty acids (VFAs), acetate, propionate, butyrate and valerate in the colon of milk-supplemented versus non-supplemented piglets at 21 days of age (weaning). Volatile fatty acids are fermentation end-products of the colonic microbiota, and the higher concentrations in the milk-supplemented pigs reflect a change in the composition of the colonic microbiota, which is likely explained by the high total dietary fibre content of the milk used in this study compared to sow milk. However, no microbiome analysis was conducted in this study.

It would appear that supplementing suckling piglets with milk from 1 to 4 days after birth until weaning can increase the weaning weight [62,63,65,67,68,71,72] and the number of piglets weaned [63,64] and reduce mortality pre-weaning [63,69]. The benefit of pre-weaning milk supplementation on intestinal maturation and microbiota after weaning is not consistent and likely linked to milk composition [62,74]. Although not extensively studied, some studies report increased post-weaning growth in response to providing a liquid milk replacer to suckling pigs [63,67,68].

**Table 3:** Effect of pre-weaning milk replacer supplementation on pre-weaning and post-weaning piglet growth and health. Litters provided with milk replacer are compared to litters not provided with milk replacer, unless otherwise stated (modified from Middelkoop, [21]).

SA ^1^ (Days)	WA ^2^ (Days)	Pattern of Provision	Pre-Weaning Effects (d0 = Birth)				Post-Weaning Effects (d0 = Weaning)				Reference
Litter size			Supplemental Milk Intake	ADG ^3^	Weaning Weight	Other	ADFI ^4^	ADG	FCR ^5^	Other	
1	21	Ad libitum	2.5 L of milk/pig (375 g DM ^6^ cool season) 9.9 L of milk/pig (1.49 kg DM warm season)	NA ^7^	↑	↑ total litter weight =mortality ↑ glucose, IGF-I ^8^ and thyroxine in serum at weaning	NA	NA	NA	NA	[65]
10.4											
4	28	Ad libitum	4.76 L of cow’s milk/pig; 10.96 L artificial milk/pig (200 g total solids/L)	=from d0 to d14 ↑ from d14 to 28 ↑ from d0 to 28	↑	NA	NA	NA	NA	NA	[71]
12											
10	20	Ad libitum	3.9 L of milk/pig (200 g of skim milk powder/L)	↑	↑	NA	↑ from d0 to d21	↑ from d0 to d21	NA	↑ weight on d21	[67]
12											
3	21	Ad libitum	1000 g of milk powder/pig	=	↑	↘ % mortality ↗ number weaned	↑ from 25 to 65 kg (grower period)	↑ from 25 to 65 kg (grower period)	=	reached slaughter weight 3 days earlier	[63]
12											
3	26	Ad libitum	13.8 mL to 10.35 L of milk/pig (winter); 43.7 mL to 17.25 L of milk/pig (summer) (150 g powder/L of water)	=	↑	=% mortality =% medicated piglets	=from d0 to 42	=from d0 to d42	=from d0 to 42	=% mortality =% medicated pigs	[72]
10 to 11											
4	21	From 8:00 to 16:00 h daily	NA in Trial 1 (late fall) 22 g of milk powder/pig in Trial 2 (summer)	=(Trial 1) ↑(Trial 2)	=(Trial 1) ↑(Trial 2)	↘ % mortality (Trial 2)	NA	↑ d21 to d54 (trial 1) =(trial 2)	NA	=carcass weight, back fat thickness, dressing percentage	[69]
10											
1	28	Twice a day or as needed	3.86 L/pig or 138 mL/pig/day (150 g of powder/L of water)	=	=	=% mortality ↑ antibiotic treatments	NA	=from d0 to d21, d21 to d72, d72 to 115	NA	=% mortality	[66]
11 to 12											
2	28	Ad libitum	520 g of powder/pig (20 g/pig/day)	=	=	↑ number weaned ↑ total litter weight =mortality, diarrhoea ↓ treatment of facial lesions	NA	NA	NA	NA	[64]
16.8											
2	21	Twice a day from 7:00 to 8:00 h and from 15:00 to 16:00 h	From d0–d7: 75 g DM ^6^ (litter/day) From d7–d14: 225 g DM (litter/day) From d14–21: 773 g DM (litter/day)	NA	↑	↑ IGF-1 ^8^ gene expression on d21 in jejunum mucosa ↑ small intestine weight on d21 ↑ crypt depth and ↓ villus height: crypt depth ratio in the ileum on d21 ↑ VFA ^11^ in the colon on d21	NA	NA	NA	NA	[62]
13 to 14											
22	27	200 mL/pig per day	172.5 g of creep/pig	↑	↑	NA	↑ from d0 to d14 ↑ from d14 to d28	↑ from d0 to d14 ↗ from d14 to d28	=from d0 to d14 ↑ from d14 to d28	NA	[68]
NA											
4	28	Ad libitum	NA	=	NA	At d28 in colon: =bacterial diversity ^9^ =bacterial species richness ^10^ ↑ VFA ↓ *Lactobacillus, Clostridium XI, Blautia, Clostridium sensu stricto, Escherichia* ↑ *Paraprevotella* ↗ *Ruminococcus, Clostridium XIVa and IV, Succiniclasticum* ↑ TLR4 ^12^ gene expression, ↓ IL-6 ^13^ gene expression in mucosa	=from d0 to d7	=from d0 to d7	NA	↘ diarrhea frequency	[76]
8											
4	28	Ad libitum access, provision of fresh milk at 9:00 and 19:00 h	NA	=	NA	=villus height, crypt depth in jejunum on d28 ↓ lactase activity and ↑ sucrase activity in jejunum	=	=	NA	In jejunum on d7: =villus height, crypt depth ↑maltase activity ↑ *Lactobacillus* ↓ *Streptococcus*	[74]
8											
7	21	Ad libitum access	NA	↑	↑	↓ diarrhoea At d21, in jejunum: ↑ bacterial species richness ^14^ =bacterial diversity ↓ *Romboutsia*, *Actinobacillus*, *Bacteroides* and *Lactobacillus*	NA	NA	NA	NA	[75]
NA											
1	28	From 15:00 h on day 1 until weaning	For all piglets alive: From d1 to d12, 1.67 L/pig or 125 mL/pig/day) From d12 to d28, 3.2 L/pig or 200 mL/pig/day (150 g of powder/L of water)	NA	↑ in litters of 17 piglets on d1 =in litters of 14 piglets on d1	↓ risk of piglets dying	NA	NA	NA	NA	[77]
14 or 17											
1	28	From 15:00 h on day 1 until weaning	NA	=	=	=body fat and body protein content	NA	NA	NA	NA	[78]
14 or 17											

↑ Significant increase; ↗ tendency to increase; ↓ Significant decrease; ↘ tendency to decrease; = No difference; ^1^ SA: start age of supplementation; ^2^ WA: weaning age; ^3^ ADG: average daily gain; ^4^ ADFI: average daily feed intake; ^5^ FCR: feed conversion ratio; ^6^ DM: dry matter; ^7^ NA: not applicable; ^8^ IGF-1: insulin-like growth factor 1; ^9^ Shannon and Simpson; ^10^ observed species and Chao1; ^11^ VFA: volatile fatty acids, ^12^ TLR4: Toll-like receptor 4;^13^ IL-6: interleukin 6; ^14^ ACE and Chao1.

#### 3.1.3. Provision of Supplemental Liquid Feed Pre-Weaning

The provision of supplemental milk pre-weaning can increase pre-weaning DMI and growth and reduce pre-weaning mortality, as outlined in Section 3.1.2. However, it does little to expose piglets to the plant-based ingredients that they will encounter in the dry diets fed post-weaning. A solution to this is to provide suckling piglets with liquid feed pre-weaning (i.e., dry feed in a gruel form or an enriched milk containing plant-based compounds). Few studies to date have compared the effect of providing supplementary liquid feed to suckling piglets, with dry creep feeding and/or with no creep feeding (dry or liquid). One such study by Lawlor et al. [79] supplemented a liquid mixture of milk and feed to suckling piglets from 12 days of age to weaning, with creep-fed litters only standardised at eight piglets. In this study, creep feeding the liquid mixture and standardising litters at eight piglets increased the weaning weight by 7%. However, the authors concluded that the increase in weaning weight was most likely achieved due to the reduced number of suckling pigs per sow. In a recent study conducted by our group, Arnaud et al. [73] supplemented suckling piglets with a liquid mixture of milk with an increasing proportion of starter feed from day 3 of age to day 28 (weaning). We did not observe an increase in weaning weight when suckling piglets were supplemented with the liquid mixture. However, pigs supplemented with the liquid mixture pre-weaning had a higher BW at slaughter (~+2 kg) than non-supplemented pigs ([73], unpublished). These results can be explained by a better intestinal structure 7 days post-weaning (+17% in villus height in the ileum), which most likely positively influenced nutrient absorption ([73], unpublished). Kobek-Kjeldager et al. [70] supplemented milk to suckling piglets from day 2 to 12 of lactation, followed by liquid feed from day 12 to weaning. This trial also compared two different weaning ages (day 24 or 35). Providing the liquid diet before weaning was found to shorten the latency period to first feed consumption post-weaning but had no impact on the latency to first water consumption following weaning. Interestingly, a change in feeding behaviour was observed at the transition from supplementary milk to liquid feed on day 12, with a reduction in the number of feeding bouts observed the day following the diet change.

Some studies demonstrated a benefit to supplementing liquid creep feed in comparison to dry creep feed in terms of increased pre-weaning ADFI [80,81]. Martins et al. [81] observed that pigs supplemented with a gruel feed (pre-gelatinised rice, micronised soybean and whey mixed with water at a 1:1 ratio) from day 3 of age to day 21 (weaning) had a ~566% higher ADFI during the first days of supplementation (day 3 to 7) than pigs supplemented with dry creep feed. Similarly, Byrgesen et al. [80] showed that pigs supplemented with liquid creep from day 10 of age to day 28 (weaning) had ~270% higher dry matter disappearance during the first week of supplementation (day 10 to 18) than pigs supplemented with dry creep. However, these studies found no increase in pre- and post-weaning ADG in response to the pre-weaning supplementation of liquid creep feed. Despite higher intakes, Byrgesen et al. [80] and Martins et al. [81] found that the weaning weight in piglets offered liquid creep feed did not differ from piglets offered dry creep feed. This could have been due to the higher number of piglet eaters observed in litters offered dry creep feed compared to litters offered liquid creep feed, even though piglets supplemented with liquid creep had a higher average intake. In the study by Byrgesen et al. [80], suckling pigs supplemented with dry creep feed were 9.6% heavier on day 61 post-weaning compared to pigs fed liquid creep feed during the suckling period. Furthermore, Martins et al. [81] showed that suckling pigs supplemented with dry creep feed had less variation in BW on day 133 post-weaning and a similar slaughter weight compared to pigs fed gruel creep feed during the suckling period. On the contrary, Arnaud et al. [73] found that pigs which were offered a liquid mixture of milk and starter diet pre-weaning were 1.6% heavier at slaughter (157 days of age) than pigs offered dry creep pre-weaning.

In a recent study, Amdi et al. [82] compared the growth and intestinal morphology and function of piglets fed a milk replacer to that of piglets fed the same milk replacer with added wheat from day 3 to 25 post-farrowing. No treatment differences were found for weaning weight, jejunal morphology (villus height, crypt depth and villus height to crypt depth ratio) and intestinal gene expression. However, an increase in the activity of sucrase and maltase in the small intestine was found just prior to weaning (~25 days of age) in response to the addition of wheat to the liquid milk. These enzymes are important for the digestion of vegetable-based ingredients, and an increase in their activity at weaning suggests that these pigs should be better equipped to digest ingredients in the normally dry diet fed post-weaning. In another study, enzyme activities just before weaning (~25 days of age) were compared between piglets offered liquid creep feed and piglets offered dry creep feed [80]. Here, the activities of sucrase and maltase in the proximal part of the small intestine were highest in piglets supplemented with dry creep even though DMI was highest when liquid creep feed was provided. Therefore, it is possible that the form of the creep feed (solid vs. liquid) may influence enzyme activity more than DMI [80]. This could be due to the occurrence of spontaneous fermentation in liquid creep feed, which changes its physicochemical properties and its effect on the GIT.

From these studies, it appears that supplementing liquid creep feed instead of dry creep feed can increase the pre-weaning ADFI [80,81]. In addition, supplementing liquid creep feed instead of milk can improve intestinal enzyme maturation [80,82]. However, increases in liquid creep feed intake and changes in intestinal function do not always result in increased growth pre- and post-weaning due to the low number of piglets consuming creep feed within litters.

### 3.2. Other Pre-Weaning Strategies to Stimulate Earlier Enzyme Production

Van den Borne et al. [83] found a positive correlation between pancreatic enzyme secretion and pig growth during the suckling period. During the suckling period, the intestinal tract is well adapted for the digestion and absorption of maternal milk. At weaning, the transition from maternal milk to solid feed leads to a remodelling of the GIT. This includes a switch in enzyme production; for example, within the intestinal brush-border disaccharidases, production switches from lactase to sucrase and maltase. Similarly, enzymes with proteolytic activity are found in relatively low concentrations during the suckling period [84,85]. Studies suggest that intestinal tract remodelling can be accelerated when exogenous enzymes are administered to suckling mammals [86,87,88]. Prykhodko et al. [87] observed an increase in gastric secretion and a switch in intestinal disaccharidases, with a decrease in lactase and an increase in maltase and sucrase in the proximal part of the small intestine, in rats supplemented with pancreatic enzymes (amylase, protease and lipase extracted from porcine pancreas) or pancreatic-like enzymes (microbially-derived alpha-amylase, proteinase and lipase). Enzyme supplementation also increased amylase and trypsin production in the pancreas [87]. Słupecka et al. [86] observed that supplementing suckling piglets twice a day for a week with porcine pancreatic enzymes increased villus height, reduced crypt depth and increased adult-type enterocyte appearance in the distal jejunal epithelium on day 16 after birth (1 day after the end of the treatment). Adult-type enterocytes are enterocytes that have differentiated into those with an absorptive function. In the same study, supplementing pigs with a complex of microbially-derived amylase, protease, and lipase also increased adult-type enterocyte appearance but decreased villus height and crypt depth on day 16 after birth, which could indicate a reduced tolerance to supplementation with exogenous enzymes. The increase in adult-type enterocyte appearance could indicate an early maturation of the intestinal epithelium. In another study by Prykhodko et al. [88], supplementing suckling pigs with a complex of microbially-derived amylase, protease and lipase once or twice between 7 and 14 days post-partum increased BW and improved the feed conversion ratio (FCR) during the grow–finishing period. In the same study, pigs supplemented with enzymes also reached the target slaughter weight earlier than non-supplemented pigs [88]. From these findings, it appears that pancreatic and microbially-derived enzyme supplementation of piglets during the suckling period may benefit lifetime growth, due to earlier maturation of the GIT. In addition, Prykhodko et al. [88] observed a decrease in nitrogen excretion per kilogram BW gain in pigs supplemented with enzymes prior to weaning compared with non-supplemented pigs, suggesting that enzyme supplementation of suckling piglets could also help in reducing the environmental impact of pig production.

To our knowledge, only two studies investigated the effect of supplementing suckling piglets with a cocktail of enzymes on pig growth up to slaughter and on intestinal structure and function pre-weaning. Therefore, there is a need for additional studies investigating the effects of supplementing suckling piglets with a enzyme cocktailS on intestinal structure and function post-weaning and pig growth up to slaughter to confirm the benefit of using this strategy on commercial farms and to understand the underlying mechanisms of action.

### 3.3. Other Pre-Weaning Strategies to Stimulate Gut Structure and Function

Beneficial effects on pig growth in response to supplementing weaned pigs with glutamine or glutamate have been demonstrated [89], as well as benefits in terms of feed efficiency [90], intestinal function [89,91] and structure [92] and reduced incidence of diarrhoea [89,93]. Glutamine and glutamate are the most abundant protein-bound amino acids in sow’s milk [94]. Sow’s milk is also rich in free glutamine, which increases in concentration from 0.1 mM on day 1 of lactation to 3.4 mM on day 29 of lactation [94]. Studies suggest that glutamine and glutamate are major fuels for enterocytes in the piglet small intestine [95]. Until recently, glutamine and glutamate were not considered essential amino acids (i.e., ones that need to be supplemented in the diet as they cannot be synthetised by the pig from a metabolic intermediate). However, additional functions have recently been assigned to amino acids, and some, including glutamine and glutamate, are now considered essential at key times (as reviewed by Watford et al. [95]). Glutamine is synthetised from glutamate and ammonia via the action of glutamine synthetase [95]. Glutamate can be synthetised via the action of glutaminase, which degrades glutamine that enters into the mitochondria into glutamate and ammonia [95]. Glutamine can be metabolised into purines and pyrimidines for the synthesis of nucleotides to support cell proliferation, while glutamate cannot [95]. For this reason, this review focuses on glutamine supplementation of suckling piglets.

Few studies to date have investigated the effects of glutamine supplementation of suckling piglets on their growth and intestinal health pre-and post-weaning. Haynes et al. [96] observed a 12% increase in growth in piglets orally supplemented with glutamine (dissolved in 20 mL of water at a concentration of 3.42 mmol/kg BW or 0.5 g/kg BW) twice daily from day 7 to 14 of age compared with piglets receiving oral treatment with alanine or water. It is important to note that the dose is critical with regard to piglet growth. Indeed, a preliminary study conducted by the same authors showed that oral supplementation with glutamine twice daily from day 7 to 21 of age at a concentration of 6.84 mmol/kg BW (i.e., 1 g of glutamine/kg BW) reduced piglet daily weight gain by 19%. Although not explained by the authors, excess glutamine can generate ammonia, which is not fully excreted by the kidneys and can therefore cause adverse effects. As the supplementation was performed twice daily, the piglets received in total 2 g of glutamine/kg BW daily, and it is likely that this rate of supplementation was above the detoxifying capacity of the animal. In the study by Haynes et al. [96], growth was also enhanced in piglets supplemented with glutamine following a lipopolysaccharide (LPS) challenge. Glutamine administration reduced the rectal temperature by 0.5 °C, improved intestinal structure (increased villus height in the jejunum) and ameliorated intestinal injury in piglets following LPS challenge [96]. In addition, they demonstrated that the addition of L-glutamine to the medium of cultured neonatal enterocytes prevented LPS- or hydrogen peroxide-induced cell death [96]. Noticeably, this effect was specific to the L-isomer. These findings support the role of L-glutamine in preventing intestinal damage in endotoxin-infected piglets, suggesting that it may have a role to play in protection against infection with Gram-negative pathogens such as enterotoxigenic *Escherichia coli*, one of the main causes of PWD. To our knowledge, L-glutamine supplementation of creep feed has only been investigated in one study to date [97]. Cabrera et al. [97] demonstrated that supplementing creep feed and nursery feed (fed after weaning) with 1% glutamine improved intestinal structure 7 days post-weaning and post-weaning FCR. However, as the supplementation was continued after weaning, it is difficult to identify the contribution of creep feed supplementation with glutamine on the positive outcomes observed post-weaning. In addition, the authors did not observe an effect of pre-weaning glutamine supplementation on weaning weight.

There is growing interest in L-glutamine supplementation for low-birth-weight (LBW) piglets as a means of improving intestinal health. Low-birth-weight piglets have higher mortality rates [98] and impaired small intestinal morphology and function [99] compared with normal-birth-weight (NBW) piglets. Few studies have investigated the effects of oral supplementation with glutamine at 1 g/kg of BW/day in suckling pigs. Li et al. [100] observed a 14.7% increase in milk intake and a 7.5% increase in BW in LBW piglets on days 11–12 post-partum when supplemented with glutamine at 1 g/kg of BW/day from day 1 via oral gavage. In both piglet categories (LBW and NBW), Schulze Holthausen et al. [101] noted a higher number of CD3+ intraepithelial lymphocytes in colon tissue and a tendency for an increase in CD3+ intraepithelial lymphocytes in the lamina propria in piglets supplemented with glutamine. A higher number of CD3+ intraepithelial lymphocytes may indicate a more mature intestinal immune system, as these cells have a signalling role in the defence of the intestinal epithelium.

During the neonatal period, gains in protein mass in skeletal muscle contribute most to growth [102]. Glutamine plays an important role in skeletal muscle development in piglets, and therefore subsequent growth [103]. Growth can be impaired in LBW piglets, which makes glutamine supplementation an interesting strategy to support their development. A recent study by Zhao et al. [104] demonstrated that glutamine can increase cell proliferation in the muscle of LBW piglets. In support of this, Zhao et al. [105] found larger muscle fibres in glutamine-supplemented piglets. However, the authors noted that the effects on skeletal muscle were minor in both studies [104,105].

In summary, the provision of glutamine at 1 g/kg of BW/day via oral gavage to piglets for 7 to 10 days before weaning can benefit piglet intestinal immunity [96,101] and growth [96,100]. However, additional work should be conducted to assess how to implement this strategy at commercial farm scale (e.g., adding glutamine to creep feed or liquid milk). Future work should also aim to study the effect of pre-weaning glutamine supplementation on post-weaning pig growth.

### 3.4. Other Pre-Weaning Strategies to Modulate Gut Microbiota

During recent decades, a number of strategies have been developed to modulate the composition of microbial (mainly bacterial) populations within the porcine gut, with the aim of improving pig growth and health. In particular, shaping the pig intestinal microbiota early in life has the potential to influence lifetime growth and health. Among the strategies investigated to date, the use of probiotics and prebiotics has been most researched, with a range of different microorganisms and compounds used across studies. Probiotics are “live microorganisms that, when administered in adequate amounts, confer a health benefit on the host” [106]. Probiotic administration to sows during gestation and/or lactation has been shown to improve colostrum/milk quality and quantity and to modulate the piglet gut microbiome, which are likely some of the mechanisms by which maternal probiotic supplementation benefits piglet growth and health [107,108]. A study from our group, among others, has demonstrated probiotic transfer from sows to suckling piglets, proving that maternal probiotic administration can be an effective means of early-life inoculation of piglets [109]. To our knowledge, our study is also the only one to date to show lifetime benefits in the offspring of probiotic-supplemented sows, namely improved growth during the finisher period and increased carcass weight at slaughter [109]. Studies also demonstrate that the administration of probiotics directly to suckling piglets can accelerate the response to enterotoxigenic *E. coli* challenge post-weaning [110] and can increase pre-weaning and early post-weaning pig growth, possibly via gut microbiota/immune modulation [111,112].

A prebiotic is “a substrate that is selectively utilised by host microorganisms conferring a health benefit” [113]. Some substances are well-accepted prebiotics (galacto-oligosaccharides (GOS), fructo-oligosaccharides (FOS), inulin and lactulose), while others are considered candidates [3,114]. Similar to probiotics, prebiotic administration to sows during gestation and/or lactation has been shown to improve colostrum quality and quantity [115,116] and to modulate piglet microbiome [115], and these effects are sometimes accompanied by improved piglet growth pre-weaning [116]. Prebiotic administration to suckling piglets has also been shown in a study by Alizadeh et al. [117] to modulate intestinal microbiome and to improve intestinal structure, although pig growth was not improved. However, this study used pigs as a model for humans, and the measurement of production parameters was not one of the main objectives. Recently, the concept of postbiotics has emerged. A postbiotic is defined as a “preparation of inanimate microorganisms and/or their components that confers a health benefit on the host” [118]. Zhong et al. [119] reviewed the use of postbiotics in livestock, and from this, it appears that only one study to date has administered postbiotics to suckling piglets. In that study, Busanello et al. [120] demonstrated that the administration of inactivated *Lactobacillus* pre-weaning increased feed intake and growth post-weaning but did not impact faecal counts of lactic acid bacteria or coliform; however, a full microbiome analysis was not conducted. The stability and safety of postbiotics make them interesting alternatives to probiotics [119].

## 4. Conclusions

Several management and nutritional strategies can be implemented to increase piglet growth to weaning and consequently improve subsequent post-weaning outcomes. Such strategies are particularly important considering recent bans on the use of pharmacological levels of ZnO and in-feed antibiotics, and continued increases in litter size. Most pre-weaning management and nutrition studies only record post-weaning growth in pigs for a limited period of time, if at all. Consequently, there is limited information on how these strategies influence lifetime growth and health in pigs, and therefore, on their economic impact. Some of the pre-weaning interventions examined in this review are inexpensive and easily implemented (e.g., split-suckling or post-partum provision of analgesia to sows). However, in some cases, additional work is needed to determine their effect on post-weaning pig growth and health. For solid creep feeding in the farrowing house, the available data suggest inconsistent effects on post-weaning pig growth due to often low and variable creep feed intake. Providing milk or liquid feed as a creep provides an opportunity to increase DMI and the proportion of eaters per litter. In addition, milk/liquid feed could be an effective route for the early-life administration of feed additives (e.g., enzymes; L-glutamine; and pro-, pre- and post-biotics). However, liquid feeding of suckling piglets in farrowing rooms needs to be conducted hygienically and requires substantial financial investment. In summary, there are many nutritional and management approaches that can be recommended to improve the pre- and post-weaning growth and health of piglets raised in large litters. However, solutions should be selected and combined on a case-by-case basis to suit the particular production system.

## Figures and Tables

**Table 1:** Pre-weaning nutritional and management strategies to improve growth and health of piglets raised in large litters.

Area	Strategies	Review
Sow management	Provision of pain relief	[16,17]
	Provision of oxytocin to prolong the colostral phase	[18]
	Optimisation of the farrowing environment (e.g., hygiene, temperature, noise and provision of substrates such as straw)	[18,19]
	Farrowing supervision and assistance	[17,18,19]
Sow nutrition	Dietary interventions during lactation: ○ L-glutamine; ○ Spray-dried plasma (porcine or bovine); ○ Polyunsaturated fatty acids (Omega 3 and 6); ○ Vitamin E and selenium; ○ Probiotics (e.g., *Bacillus*); ○ Prebiotics (e.g., fructo oligosaccharides).	[20] [17] [17,20] [17] [17] [18]
Piglet management	Split-suckling	[18,19,20]
	Cross-fostering	[17,18,19,20]
	Nurse sows	[18,19]
	Optimised farrowing environment: ○ Supplementary heat; ○ Provision of substrates such as straw; ○ Optimisation of feeder type.	[18] [18] [21]
	Increasing weaning age	[3,20,22,23]
	Split-weaning	[24]
	Intermittent suckling	[20]
	Artificial rearing	[18,19]
	Socialisation (mixing piglets before weaning)	[20]
Piglet nutrition	Injection of glucose (energy booster)	[18]
	Oral supplementation: ○ Amino acids (e.g., glutamine); ○ Colostrum (bovine, sow or replacer); ○ Medium chain fatty acids; ○ Prebiotics (e.g., inulin, β-glucans and oligosaccharides); ○ Faecal microbiota transplant; ○ Nucleotides.	[22] [17,18] [18] [22] [25] [17]
	Creep feeding: ○ Supplementary solid feed; ○ Supplementary liquid feed (mix of feed and water or milk); ○ Supplementary milk.	[20,22,26] [17,22] [17,18,19,22]
	Dietary intervention through creep feed: ○ Amino acids (e.g., glutamine); ○ Fat sources rich in medium chain fatty acids; ○ Fibre sources; ○ Flavours; ○ Prebiotics (e.g., oligofructose); ○ Synbiotics; ○ Probiotics (e.g., yeast).	[22] [22] [22] [26] [22] [22] [22]

**Table 2:** Overview of the efficacy of non-steroidal anti-inflammatory drugs administered to sows in sows and piglets during the periparturient period. Treated sows were compared with untreated sows, unless otherwise stated (modified from Schoos et al. [16]).

Medication	Dose	Route of Administration	Timing	Effects on Sows	Effects on Piglets	Reference
Meloxicam	0.4 mg/kg BW ^1^	Intramuscular	~90 min post-partum	↓ time lying during day 3 post-partum =FI ^2^ =RT ^3^	=mortality ↑ ADG ^4^ of low birth weight piglets (<1180 g) from multiparous sows	[42]
Meloxicam	0.4 mg/kg BW	Intramuscular	~12 h post-partum	=RT	=mortality ↑ litter size at weaning ↗ weight gain in litter of 11–13 pigs	[43]
Meloxicam	0.4 mg/kg BW	Oral gavage	Beginning of farrowing	NS ^5^	=mortality ↑ ADG and weaning weight ↑ IgG ^6^ in serum on day 1 and 2	[39]
Meloxicam/ paracetamol	0.4/30 mg/kg BW	Oral gavage	Once a day for 7 days from day 113 of gestation (sows with PDS ^7^)	=RT paracetamol ↓ RT vs. meloxicam	=mortality =ADG	[41]
Meloxicam	0.4 mg/kg BW	Oral gavage	Beginning of farrowing	↗ colostrum IgA ^8^ and IgG	=mortality ↗ ADG from day 9 to weaning ↑ IgA in serum on day 1 and 9 ↗ IL-2 ^9^ and IL-4 ^9^ in serum on day 9	[40]
Meloxicam	0.4 mg/kg BW	Intramuscular	~2 h post-partum	=back fat at weaning ↓ body weight at weaning	↗ colostrum intake =mortality ↓ antibiotics/anti-inflammatories ↑ ADG and weaning weight ↑ slaughter weight	[51]
Meloxicam/Flunixin (no untreated sows)	0.4/2 mg/kg BW	Intramuscular	1.5–24 h post-clinical PDS signs	FI: meloxicam = flunixin RT: meloxicam = flunixin	Mortality: meloxicam < flunixin ADG: meloxicam = flunixin	[50]
Flunixin/ Metamizole (no untreated sows)	0.5/50 mg/kg BW	Intramuscular	End of parturition +24 h later if needed	Flunixin: ↓ RT (day 1 vs. day 3 post-partum) Metamizole: =RT (day 1 vs. day 3 post-partum)	NS	[44]
Paracetamol	20 mL of paracetamol (400 mg/mL)	Over the feed divided over two meals	6 days from 3 days before farrowing to 2 days post-partum	=RT ↑ back fat at weaning	=mortality =ADG =IgG in serum on day 1	[49]
Ketoprofen	3 mg/kg BW	Intramuscular	During 3 days post-partum	↓ incidence of feed refusal ↑ back fat in week 2 ↓ constipation duration	=ADG	[46]
Ketoprofen	3 mg/kg BW	Intramuscular	Within 12 h post-partum	NS	↓ mortality ↑ litter size at weaning	[47]
Ketoprofen	1 mg/kg BW	Intramuscular	Within 12 h post-partum	=back fat at weaning ↓ RT	↘ mortality =weight gain per litter	[45]
Ketoprofen	3 mg/kg BW	Intramuscular	1.5 h post-partum	=putative pain behaviours =salivary cortisol =C-reactive protein =cytokines	NS	[48]

^1^ BW, body weight; ^2^ FI, feed intake; ^3^ RT, rectal temperature; ^4^ ADG, average daily gain; ^5^ NS, not studied; ^6^ IgG, immunoglobulin G; ^7^ PDS, postpartum dysgalactia syndrome and mastitis; ^8^ IgA, immunoglobulin A; ^9^ IL-2(4), Interleukin-2(4); ↑ significant increase; ↗ tendency to increase; ↓ significant decrease; ↘ tendency to decrease; = no difference.

## Data Availability

Not applicable.

## References

[1] Colson V., Martin E., Orgeur P., Prunier A. (2012). Influence of housing and social changes on growth, behaviour and cortisol in piglets at weaning. Physiol. Behav..

[2] Klobasa F., Werhahn E., Butler J.E. (1987). Composition of sow milk during lactation. J. Anim. Sci..

[3] Lawlor P.G., Gardiner G.E., Goodband R.D., Farmer C. (2020). 10. Feeding the weaned piglet. The Suckling and Weaned Piglet.

[4] Heo J.M., Opapeju F.O., Pluske J.R., Kim J.C., Hampson D.J., Nyachoti C.M. (2013). Gastrointestinal health and function in weaned pigs: A review of feeding strategies to control post-weaning diarrhoea without using in-feed antimicrobial compounds. J. Anim. Physiol. Anim. Nutr..

[5] Tang X., Xiong K., Fang R., Li M. (2022). Weaning stress and intestinal health of piglets: A review. Front. Immunol..

[6] Gresse R., Chaucheyras-Durand F., Fleury M.A., Van de Wiele T., Forano E., Blanquet-Diot S. (2017). Gut microbiota dysbiosis in postweaning pglets: Uderstanding the kys to halth. Trends Microbiol..

[7] Canibe N., Højberg O., Kongsted H., Vodolazska D., Lauridsen C., Nielsen T.S., Schönherz A.A. (2022). Review on preventive measures to reduce post-weaning diarrhoea in piglets. Animals.

[8] Bednorz C., Oelgeschläger K., Kinnemann B., Hartmann S., Neumann K., Pieper R., Bethe A., Semmler T., Tedin K., Schierack P. (2013). The broader context of antibiotic resistance: Zinc feed supplementation of piglets increases the proportion of multi-resistant Escherichia coli in vivo. Int. J. Med. Microbiol..

[9] Iramiot J.S., Kajumbula H., Bazira J., Kansiime C., Asiimwe B.B. (2020). Antimicrobial resistance at the human–animal interface in the Pastoralist Communities of Kasese District, South Western Uganda. Sci. Rep..

[10] (2019). Regulation (EU) 2019/4 of the European Parliament and of the Council of 11 December 2018 on the Manufacture, Placing on the Market and Use of Medicated Feed, Amending Regulation (EC) No 183/2005 of the European Parliament and of the Council and Repealing Council Directive 90/167/EEC; Official Journal of the European Union.

[11] (2017). Zinc Oxide Article-35 Referral. Zinc Oxide Article-35 Referral—Annex I, II. EMEA/V/A/118. https://www.ema.europa.eu/en/documents/referral/zinc-oxide-article-35-referral-annex-iii_en.pdf.

[12] Oliviero C. (2022). Offspring of hyper prolific sows: Immunity, birthweight, and heterogeneous litters. Mol. Reprod. Dev..

[13] King R. (2000). Factors that influence milk production in well-fed sows. J. Anim. Sci..

[14] Devillers N., Le Dividich J., Prunier A. (2011). Influence of colostrum intake on piglet survival and immunity. Animal.

[15] Collins C.L., Pluske J.R., Morrison R.S., McDonald T.N., Smits R.J., Henman D.J., Stensland I., Dunshea F.R. (2017). Post-weaning and whole-of-life performance of pigs is determined by live weight at weaning and the complexity of the diet fed after weaning. Anim. Nutr..

[16] Schoos A., Devreese M., Maes D.G. (2019). Use of non-steroidal anti-inflammatory drugs in porcine health management. Vet. Rec..

[17] Blavi L., Solà-Oriol D., Llonch P., López-Vergé S., Martín-Orúe S.M., Pérez J.F. (2021). Management and feeding strategies in early life to increase piglet performance and welfare around weaning: A review. Animals.

[18] Farmer C., Edwards S.A. (2022). Review: Improving the performance of neonatal piglets. Animal.

[19] Baxter E.M., Schmitt O., Pedersen L.J., Farmer C. (2020). 3. Managing the litter from hyperprolific sows. The Suckling and Weaned Piglet.

[20] Wensley M.R., Tokach M.D., Woodworth J.C., Goodband R.D., Gebhardt J.T., DeRouchey J.M., McKilligan D. (2021). Maintaining continuity of nutrient intake after weaning. I. Review of pre-weaning strategies. Transl. Anim. Sci..

[21] Middelkoop A. (2020). Foraging in the Farrowing Room. Ph.D. Thesis.

[22] Huting A.M.S., Middelkoop A., Guan X., Molist F. (2021). Using nutritional strategies to shape the gastro-intestinal tracts of suckling and weaned piglets. Animals.

[23] Edwards S., Turpin D.L., Pluske J., Farmer C. (2020). 9. Weaning age and its long-term influence on health and performance. The Suckling and Weaned Piglet.

[24] De Vos M., Che L., Huygelen V., Willemen S., Michiels J., Van Cruchten S., Van Ginneken C. (2014). Nutritional interventions to prevent and rear low-birthweight piglets. J. Anim. Physiol. Anim. Nutr..

[25] Canibe N., O’Dea M., Abraham S. (2019). Potential relevance of pig gut content transplantation for production and research. J. Anim. Sci. Biotechnol..

[26] Tokach M., Scher Cemin H., Sulabo R., Goodband R., Farmer C. (2020). Feeding the suckling pig: Creep feeding. The Suckling and Weaned Piglet.

[27] Rutherford K., Baxter E., D’Eath R., Turner S., Arnott G., Roehe R., Ask B., Sandøe P., Moustsen V., Thorup F. (2013). The welfare implications of large litter size in the domestic pig I: Biologica factors. Anim. Welf..

[28] Hasan S., Orro T., Valros A., Junnikkala S., Peltoniemi O., Oliviero C. (2019). Factors affecting sow colostrum yield and composition, and their impact on piglet growth and health. Livest. Sci..

[29] Curtis J., Bourne F.J. (1971). Immunoglobulin quantitation in sow serum, colostrum and milk and the serum of young pigs. Biochim. Biophys. Acta.

[30] Dividich J.L., Rooke J.A., Herpin P. (2005). Nutritional and immunological importance of colostrum for the new-born pig. J. Agric. Sci..

[31] Xu R.J., Sangild P.T., Zhang Y.Q., Zhang S.H., Zabielski R., Gregory P.C., Weström B., Salek E. (2002). Chapter 5 Bioactive compounds in porcine colostrum and milk and their effects on intestinal development in neonatal pigs11This work has been supported by the Research Grants Council of the Hong Kong Special Administrative Region, China (HKU 7234/98M). Biology of Growing Animals.

[32] Quesnel H., Farmer C., Devillers N. (2012). Colostrum intake: Influence on piglet performance and factors of variation. Livest. Sci..

[33] Fraser D. (1984). The role of behavior in swine production: A review of research. Appl. Anim. Ethol..

[34] Herskin M.S., Di Giminiani P., Špinka M. (2018). 11—Pain in pigs: Characterisation, mechanisms and indicators. Advances in Pig Welfare.

[35] Kovac G., Tóthová C., Oskar N., Seidel H. (2008). Acute phase proteins during the reproductive cycle of sows. Acta Vet..

[36] European Medicines Agency—Science Medicines Health Medicines. https://www.ema.europa.eu/en/medicines.

[37] Ghlichloo I., Gerriets V. (2021). Nonsteroidal Anti-Inflammatory Drugs (NSAIDs).

[38] Chaiamnuay S., Allison J.J., Curtis J.R. (2006). Risks versus benefits of cyclooxygenase-2-selective nonsteroidal antiinflammatory drugs. Am. J. Health-Syst. Pharm..

[39] Mainau E., Temple D., Manteca X. (2016). Experimental study on the effect of oral meloxicam administration in sows on pre-weaning mortality and growth and immunoglobulin G transfer to piglets. Prev. Vet. Med..

[40] Navarro E., Mainau E., de Miguel R., Temple D., Salas M., Manteca X. (2021). Oral meloxicam administration in sows at farrowing and Its effects on piglet immunity transfer and growth. Front. Vet. Sci..

[41] Schoos A., Chantziaras I., Vandenabeele J., Biebaut E., Meyer E., Cools A., Devreese M., Maes D. (2020). Prophylactic use of meloxicam and paracetamol in peripartal sows suffering from postpartum dysgalactia syndrome. Front. Vet. Sci..

[42] Mainau E., Ruiz-de-la-Torre J.L., Dalmau A., Salleras J.M., Manteca X. (2012). Effects of meloxicam (Metacam^®^) on post-farrowing sow behaviour and piglet performance. Animal.

[43] Tenbergen R., Friendship R., Cassar G., Amezcua M., Haley D. (2014). Investigation of the use of meloxicam post farrowing for improving sow performance and reducing pain. J. Swine Health Prod..

[44] Tummaruk P., Sang-Gassanee K. (2013). Effect of farrowing duration, parity number and the type of anti-inflammatory drug on postparturient disorders in sows: A clinical study. Trop. Anim. Health Prod..

[45] Claeyé E., Beek J., Meyns T., Maes D. (2015). Effect of ketoprofen treatment in the prevention of postpartum dysgalactia syndrome in sows. Vlaams Diergeneeskd. Tijdschr..

[46] Viitasaari E., Hänninen L., Heinonen M., Raekallio M., Orro T., Peltoniemi O., Valros A. (2013). Effects of post-partum administration of ketoprofen on sow health and piglet growth. Vet. J..

[47] Homedes J., Salichs M., Sabaté D., Sust M., Fabre R. (2014). Effect of ketoprofen on pre-weaning piglet mortality on commercial farms. Vet. J..

[48] Ison S.H., Jarvis S., Hall S.A., Ashworth C.J., Rutherford K.M.D. (2018). Periparturient Behavior and Physiology: Further Insight Into the Farrowing Process for Primiparous and Multiparous Sows. Front. Vet. Sci..

[49] Kuller W., Sietsma S., Hendriksen S., Sperling D. (2021). Use of paracetamol in sows around farrowing: Effect on health and condition of the sow, piglet mortality, piglet weight and piglet weight gain. Porc. Health Manag..

[50] Hirsch A.C., Philipp H., Kleemann R. (2003). Investigation on the efficacy of meloxicam in sows with mastitis-metritis-agalactia syndrome. J. Vet. Pharmacol. Ther..

[51] Arnaud E.A., Gardiner G.E., Halpin K.M., Ribas C., O’Doherty J.V., Sweeney T., Lawlor P.G. (2023). Post-partum meloxicam administration to sows but not split-suckling increases piglet growth and reduces medicinal treatment of piglets. J. Anim. Sci..

[52] Bandrick M., Pieters M., Pijoan C., Baidoo S.K., Molitor T.W. (2011). Effect of cross-fostering on transfer of maternal immunity to Mycoplasma hyopneumoniae to piglets. Vet. Rec..

[53] Donovan T.S., Dritz S. (1996). Effects of split-nursing management on growth performance in nursing pigs. Kans. Agric. Exp. Stn. Res. Rep..

[54] Baxter E., Rutherford K., Arnott G., D’Eath R., Turner S., Jarvis S., Sandøe P., Moustsen V., Thorup F., Edwards S. (2013). The welfare implications of large litter size in the domestic pig II: Management factors. Anim. Welf..

[55] Vandaele M., Van Kerschaver C., Degroote J., Van Ginneken C., Michiels J. (2020). Piglet performance and colostrum intake in litters either or not split-suckled during the first day or during the first three days of life. Livest. Sci..

[56] Kyriazakis I., Edwards S. (1986). The effect of “split-suckling” on behaviour and performance of piglets. Appl. Anim. Behav. Sci..

[57] Donovan T.S., Dritz S.S. (2000). Effect of split nursing on variation in pig growth from birth to weaning. J. Am. Vet. Med. Assoc..

[58] Morton J.M., Langemeier A.J., Rathbun T.J., Davis D.L. (2019). Immunocrit, colostrum intake, and preweaning body weight gain in piglets after split suckling based on birth weight or birth order. Transl. Anim. Sci..

[59] Muns R., Manteca X., Gasa J. (2015). Effect of different management techniques to enhance colostrum intake on piglets’ growth and mortality. Anim. Welf..

[60] Galiot L., Lachance I., Laforest J.-P., Guay F. (2018). Modelling piglet growth and mortality on commercial hog farms using variables describing individual animals, litters, sows and management factors. Anim. Reprod. Sci..

[61] Huser J., Plush K., Pitchford W., Kennett T., Lines D. (2015). Neonatal split suckling improves survival of small piglets. Anim. Prod. Sci..

[62] De Greeff A., Resink J.W., van Hees H.M., Ruuls L., Klaassen G.J., Rouwers S.M., Stockhofe-Zurwieden N. (2016). Supplementation of piglets with nutrient-dense complex milk replacer improves intestinal development and microbial fermentation. J. Anim. Sci..

[63] Wolter B.F., Ellis M., Corrigan B.P., DeDecker J.M. (2002). The effect of birth weight and feeding of supplemental milk replacer to piglets during lactation on preweaning and postweaning growth performance and carcass characteristics. J. Anim. Sci..

[64] Pustal J., Traulsen I., Preißler R., Müller K., Beilage T., Börries U., Kemper N. (2015). Providing supplementary, artificial milk for large litters during lactation: Effects on performance and health of sows and piglets: A case study. Porc. Health Manag..

[65] Azain M.J., Tomkins T., Sowinski J.S., Arentson R.A., Jewell D.E. (1996). Effect of supplemental pig milk replacer on litter performance: Seasonal variation in response. J. Anim. Sci..

[66] Douglas S.L., Edwards S.A., Kyriazakis I. (2014). Management strategies to improve the performance of low birth weight pigs to weaning and their long-term consequences. J. Anim. Sci..

[67] Dunshea F., Kerton D.J., Eason P., King R.H. (1999). Supplemental skim milk before and after weaning improves growth performance of pigs. Crop Pasture Sci..

[68] Van Oostrum M., Lammers A., Molist F. (2016). Providing artificial milk before and after weaning improves postweaning piglet performance. J. Anim. Sci..

[69] Park B.C., Ha D.M., Park M.J., Lee C.Y. (2014). Effects of milk replacer and starter diet provided as creep feed for suckling pigs on pre- and post-weaning growth. Anim. Sci. J..

[70] Kobek-Kjeldager C., Vodolazs’ka D., Lauridsen C., Canibe N., Pedersen L.J. (2021). Impact of supplemental liquid feed pre-weaning and piglet weaning age on feed intake post-weaning. Livest. Sci..

[71] Dunshea F., Boyce J., King R. (1998). Effect of supplemental nutrients on the growth performance of sucking pigs. Aust. J. Agric. Res..

[72] Miller Y.J., Collins A.M., Smits R.J., Thomson P.C., Holyoake P.K. (2012). Providing supplemental milk to piglets preweaning improves the growth but not survival of gilt progeny compared with sow progeny. J. Anim. Sci..

[73] Arnaud E.A., Gardiner G.E., Chombart M., O’Doherty J.V., Sweeney T., Lawlor P.G. (2023). Effect of creep feeding solid starter diet, liquid milk and a liquid mixture of starter diet and milk to suckling pigs on their growth and medication usage. J. Anim. Sci..

[74] Hu P., Niu Q., Zhu Y., Shi C., Wang J., Zhu W. (2020). Effects of early commercial milk supplement on the mucosal morphology, bacterial community and bacterial metabolites in jejunum of the pre- and post-weaning piglets. Asian-Australas. J. Anim. Sci..

[75] Jin J., Jia J., Zhang L., Chen Q., Zhang X., Sun W., Ma C., Xu F., Zhan S., Ma L. (2020). Jejunal inflammatory cytokines, barrier proteins and microbiome-metabolome responses to early supplementary feeding of Bamei suckling piglets. BMC Microbiol..

[76] Shi C., Zhu Y., Niu Q., Wang J., Wang J., Zhu W. (2018). The changes of colonic bacterial composition and bacterial metabolism induced by an early food introduction in a neonatal porcine model. Curr. Microbiol..

[77] Kobek-Kjeldager C., Moustsen V.A., Theil P.K., Pedersen L.J. (2020). Effect of litter size, milk replacer and housing on production results of hyper-prolific sows. Animal.

[78] Kobek-Kjeldager C., Moustsen V.A., Pedersen L.J., Theil P.K. (2021). Impact of litter size, supplementary milk replacer and housing on the body composition of piglets from hyper-prolific sows at weaning. Animal.

[79] Lawlor P.G., Lynch P.B., Caffrey P.J., O’ Doherty J.V. (2002). Effect of pre- and post-weaning management on subsequent pig performance to slaughter and carcass quality. Anim. Sci..

[80] Byrgesen N., Madsen J.G., Larsen C., Kjeldsen N.J., Cilieborg M.S., Amdi C. (2021). The effect of feeding liquid or dry creep feed on growth performance, feed disappearance, enzyme activity and number of eaters in suckling piglets. Animals.

[81] Martins S.M.M.K., Ferrin M.O., Poor A.P., Campos G.A., Torres M.A., Weigel R.A., Strefezzi R.F., Andrade A.F.C. (2020). Gruel creep feed provided from 3 days of age did not affect the market weight and the sow’s catabolic state. Livest. Sci..

[82] Amdi C., Pedersen M.L.M., Klaaborg J., Myhill L.J., Engelsmann M.N., Williams A.R., Thymann T. (2021). Pre-weaning adaptation responses in piglets fed milk replacer with gradually increasing amounts of wheat. Br. J. Nutr..

[83] Van den Borne J.J., Weström B.R., Kruszewska D., Botermans J.A., Svendsen J., Woliński J., Pierzynowski S.G. (2007). Exocrine pancreatic secretion in pigs fed sow’s milk and milk replacer, and its relationship to growth performance. J. Anim. Sci..

[84] Pierzynowski S.G., Weström B.R., Svendsen J., Karlsson B.W. (1990). Development of exocrine pancreas function in chronically cannulated pigs during 1-13 weeks of postnatal life. J. Pediatr. Gastroenterol. Nutr..

[85] Pierzynowski S.G., Weström B.R., Erlanson-Albertsson C., Ahre’n B., Svendsen J., Karlsson B.W. (1993). Induction of exocrine pancreas maturation at weaning in young developing pigs. J. Pediatr. Gastroenterol. Nutr..

[86] Słupecka M., Woliński J., Prykhodko O., Ochniewicz P., Gruijc D., Fedkiv O., Weström B.R., Pierzynowski S.G. (2012). Stimulating effect of pancreatic-like enzymes on the development of the gastrointestinal tract in piglets. J. Anim. Sci..

[87] Prykhodko O., Pierzynowski S.G., Nikpey E., Arevalo Sureda E., Fedkiv O., Weström B.R. (2015). Pancreatic and pancreatic-like microbial proteases accelerate gut maturation in neonatal rats. PLoS ONE.

[88] Prykhodko O., Fedkiv O., Szwiec K., Botermans J., Weström B., Pierzynowski S. (2016). Early treatment with pancreatic-like microbial-derived enzymes during the preweaning period promotes growth in growing–finishing pigs. J. Anim. Sci..

[89] Teixeira A., Nogueira E., Kutschenko M., Rostagno H., Lopes D. (2014). Inclusion of glutamine associated with glutamic acid in the diet of piglets weaned at 21 days of age. Rev. Bras. Saúde Produção Anim..

[90] Wu G., Meier S.A., Knabe D.A. (1996). Dietary glutamine supplementation prevents jejunal atrophy in weaned pigs. J. Nutr..

[91] Domeneghini C., Di Giancamillo A., Bosi G., Arrighi S. (2006). Can nutraceuticals affect the structure of intestinal mucosa? Qualitative and quantitative microanatomy in L-glutamine diet-supplemented weaning piglets. Vet. Res. Commun..

[92] Molino J., Donzele J., Oliveira R., Haese D., Fortes E., Souza M.F.D.S. (2012). L-glutamine and L-glutamate in diets with different lactose levels for piglets weaned at 21 days of age. Rev. Bras. Zootec..

[93] Rezaei R., Knabe D.A., Tekwe C.D., Dahanayaka S., Ficken M.D., Fielder S.E., Eide S.J., Lovering S.L., Wu G. (2013). Dietary supplementation with monosodium glutamate is safe and improves growth performance in postweaning pigs. Amino Acids.

[94] Wu G., Knabe D.A. (1994). Free and protein-bound amino acids in sow’s colostrum and milk. J. Nutr..

[95] Watford M. (2015). Glutamine and glutamate: Nonessential or essential amino acids?. Anim. Nutr..

[96] Haynes T.E., Li P., Li X., Shimotori K., Sato H., Flynn N.E., Wang J., Knabe D.A., Wu G. (2009). L-Glutamine or L-alanyl-L-glutamine prevents oxidant- or endotoxin-induced death of neonatal enterocytes. Amino Acids.

[97] Cabrera R.A., Usry J.L., Arrellano C., Nogueira E.T., Kutschenko M., Moeser A.J., Odle J. (2013). Effects of creep feeding and supplemental glutamine or glutamine plus glutamate (Aminogut) on pre- and post-weaning growth performance and intestinal health of piglets. J. Anim. Sci. Biotechnol..

[98] Quiniou N., Dagorn J., Gaudré D. (2002). Variation of piglets’ birth weight and consequences on subsequent performance. Livest. Prod. Sci..

[99] Ayuso M., Irwin R., Walsh C., Van Cruchten S., Van Ginneken C. (2021). Low birth weight female piglets show altered intestinal development, gene expression, and epigenetic changes at key developmental loci. FASEB J..

[100] Li Z., Sciascia Q.L., Görs S., Nguyen N., Rayatdoost Baghal F., Schregel J., Tuchscherer A., Zentek J., Metges C.C. (2022). Glutamine supplementation moderately affects growth, plasma metabolite and free amino acid patterns in neonatal low birth weight piglets. Br. J. Nutr..

[101] Schulze Holthausen J., Schregel J., Sciascia Q.L., Li Z., Tuchscherer A., Vahjen W., Metges C.C., Zentek J. (2022). Effects of oral glutamine supplementation, birthweight and age on colonic morphology and microbiome development in male suckling piglets. Microorganisms.

[102] Rudar M., Fiorotto M.L., Davis T.A. (2019). Regulation of muscle growth in early postnatal life in a swine model. Annu. Rev. Anim. Biosci..

[103] Wu G., Bazer F.W., Johnson G.A., Knabe D.A., Burghardt R.C., Spencer T.E., Li X.L., Wang J.J. (2011). TRIENNIAL GROWTH SYMPOSIUM: Important roles for L-glutamine in swine nutrition and production1,2. J. Anim. Sci..

[104] Zhao Y., Albrecht E., Stange K., Li Z., Schregel J., Sciascia Q.L., Metges C.C., Maak S. (2021). Glutamine supplementation stimulates cell proliferation in skeletal muscle and cultivated myogenic cells of low birth weight piglets. Sci. Rep..

[105] Zhao Y., Albrecht E., Sciascia Q.L., Li Z., Görs S., Schregel J., Metges C.C., Maak S. (2020). Effects of oral glutamine supplementation on early postnatal muscle morphology in low and normal birth weight piglets. Animals.

[106] Hill C., Guarner F., Reid G., Gibson G.R., Merenstein D.J., Pot B., Morelli L., Canani R.B., Flint H.J., Salminen S. (2014). The International Scientific Association for Probiotics and Prebiotics consensus statement on the scope and appropriate use of the term probiotic. Nat. Rev. Gastroenterol. Hepatol..

[107] Liao S.F., Nyachoti M. (2017). Using probiotics to improve swine gut health and nutrient utilization. Anim. Nutr..

[108] Barba-Vidal E., Martín-Orúe S.M., Castillejos L. (2019). Practical aspects of the use of probiotics in pig production: A review. Livest. Sci..

[109] Crespo-Piazuelo D., Gardiner G.E., Ranjitkar S., Bouwhuis M.A., Ham R., Phelan J.P., Marsh A., Lawlor P.G. (2022). Maternal supplementation with Bacillus altitudinis spores improves porcine offspring growth performance and carcass weight. Br. J. Nutr..

[110] Hansen L.H.B., Lauridsen C., Nielsen B., Jørgensen L., Canibe N. (2022). Impact of early inoculation of probiotics to suckling piglets on postweaning diarrhoea—A challenge study with Enterotoxigenic E. Coli F18. Animal.

[111] Kiros T.G., Luise D., Derakhshani H., Petri R., Trevisi P., D’Inca R., Auclair E., van Kessel A.G. (2019). Effect of live yeast Saccharomyces cerevisiae supplementation on the performance and cecum microbial profile of suckling piglets. PLoS ONE.

[112] Xin J., Zeng D., Wang H., Sun N., Zhao Y., Dan Y., Pan K., Jing B., Ni X. (2020). Probiotic Lactobacillus johnsonii BS15 promotes growth performance, intestinal immunity, and gut microbiota in piglets. Probiotics Antimicrob. Proteins.

[113] Gibson G.R., Hutkins R., Sanders M.E., Prescott S.L., Reimer R.A., Salminen S.J., Scott K., Stanton C., Swanson K.S., Cani P.D. (2017). Expert consensus document: The International Scientific Association for Probiotics and Prebiotics (ISAPP) consensus statement on the definition and scope of prebiotics. Nat. Rev. Gastroenterol. Hepatol..

[114] Scott K.P., Grimaldi R., Cunningham M., Sarbini S.R., Wijeyesekera A., Tang M.L.K., Lee J.C.Y., Yau Y.F., Ansell J., Theis S. (2020). Developments in understanding and applying prebiotics in research and practice—An ISAPP conference paper. J. Appl. Microbiol..

[115] Hasan S., Junnikkala S., Peltoniemi O., Paulin L., Lyyski A., Vuorenmaa J., Oliviero C. (2018). Dietary supplementation with yeast hydrolysate in pregnancy influences colostrum yield and gut microbiota of sows and piglets after birth. PLoS ONE.

[116] Davis H.E., Jagger S., Toplis P., Miller H.M. (2021). Feeding β-hydroxy β-methyl butyrate to sows in late gestation improves litter and piglet performance to weaning and colostrum immunoglobulin concentrations. Anim. Feed Sci. Technol..

[117] Alizadeh A., Akbari P., Difilippo E., Schols H.A., Ulfman L.H., Schoterman M.H.C., Garssen J., Fink-Gremmels J., Braber S. (2016). The piglet as a model for studying dietary components in infant diets: Effects of galacto-oligosaccharides on intestinal functions. Br. J. Nutr..

[118] Salminen S., Collado M.C., Endo A., Hill C., Lebeer S., Quigley E.M.M., Sanders M.E., Shamir R., Swann J.R., Szajewska H. (2021). The International Scientific Association of Probiotics and Prebiotics (ISAPP) consensus statement on the definition and scope of postbiotics. Nat. Rev. Gastroenterol. Hepatol..

[119] Zhong Y., Wang S., Di H., Deng Z., Liu J., Wang H. (2022). Gut health benefit and application of postbiotics in animal production. J. Anim. Sci. Biotechnol..

[120] Busanello M., Pozza M., Pozza P., Nunes R., Sartório Chambo A.P., Eckstein I. (2015). Probiotics: Viable and inactivated cells on the performance, microflora and blood parameters of piglets. Rev. Bras. Saude Prod. Anim..

